# Refractory Anaplastic Large Cell Lymphoma Rescued by the Combination of the Second-Generation ALK Inhibitor Brigatinib, High-dose Chemotherapy and Allogeneic Stem Cell Transplantation: A Case Report and Review of the Literature

**DOI:** 10.1007/s44228-023-00038-6

**Published:** 2023-04-18

**Authors:** Giulia Caddeo, Cristina Tecchio, Matteo Chinello, Rita Balter, Ada Zaccaron, Virginia Vitale, Vincenza Pezzella, Elisa Bonetti, Marta Pillon, Elisa Carraro, Lara Mussolin, Simone Cesaro

**Affiliations:** 1 Pediatric Hematology-Oncology, Department of Mother and Child, Azienda Ospedaliera Universitaria Integrata Verona, Verona, Italy; 2 Section of Hematology and Bone Marrow Transplant Unit, Department of Medicine, Verona University Verona, Verona, Italy; 3 Department of Women’s and Children’s Health, Clinic of Pediatric Hematology-Oncology, University of Padova, Padua, Italy; 4 Pediatric Research Institute, Fondazione Città Della Speranza, Padua, Italy

**Keywords:** Anaplastic large cell lymphoma, Pediatric non-Hodgkin lymphoma, Stem cell transplant, Brigatinib, ALK lymphoma

## Abstract

The treatment of pediatric patients with refractory or relapsed anaplastic large cell lymphoma (ALCL) is still a major challenge. In addition to conventional chemotherapy and stem cell transplantation, new therapeutic options such as anti-CD30 drugs and anaplastic lymphoma kinase (ALK) inhibitors have been recently introduced in this setting. Among ALK inhibitors, only the first-generation molecule crizotinib is approved for pediatric use, while second-generation molecules, such as brigatinib, are still under investigation. Here we report the case of a 13-year-old boy diagnosed with stage IV ALCL, refractory to first-line conventional chemotherapy and second-line therapy with the anti CD30 antibody–drug conjugate brentuximab-vedotin, who finally achieved remission after a combination of conventional high-dose chemotherapy and the second-generation ALK inhibitor brigatinib. The latter was chosen for its ability to penetrate through the blood–brain barrier, due to the persistent involvement of the patient’s cerebral nervous system. The remission was then consolidated with an allogeneic hematopoietic stem cell transplantation (HSCT) from an unrelated donor using myeloablative conditioning with total body irradiation. At 24 months after HSCT, the patient is in complete remission, alive and well. An updated review regarding the use of ALK inhibitors in ALCL patients is provided.

## Introduction

Anaplastic large cell lymphoma (ALCL) represents 10–15% of non-Hodgkin lymphomas in the pediatric age. About 20–30% of pediatric patients treated according to first-line conventional chemotherapy protocols will experience disease relapse or progression, and up to half of these will succumb to the disease itself [1–3].

Currently, there is no consensus about the best management of refractory/relapsed (R/R) ALCL [4]. New therapeutic options, in particular the introduction of the anti-CD30 antibody–drug conjugate brentuximab-vedotin and anaplastic lymphoma kinase (ALK) inhibitors, are being studied in this setting, especially in high-risk patients [1, 2, 5]. Allogeneic hematopoietic stem cell transplantation (HSCT) plays an important role to consolidate remission in R/R patients, but its efficacy depends also on the pre-HSCT remission status [6–8].

Here, we report the case of a 13-year-old boy with CD30 + /ALK + ALCL who failed to achieve remission after conventional multiagent chemotherapy and experienced a rapid disease progression, despite a second-line treatment with brentuximab-vedotin, steroids and vinblastine. The patient obtained complete remission with a third-line treatment based on the combination of brigatinib, a second-generation ALK-inhibitor, and conventional high-dose chemotherapy. The remission was consolidated with allogeneic HSCT. The patient is alive and well at 24 months from transplant and 36 months from diagnosis. A review of the relevant literature regarding the use of ALK-inhibitor in ALCL patients is provided.

## Case Report

**Fig. 1:**
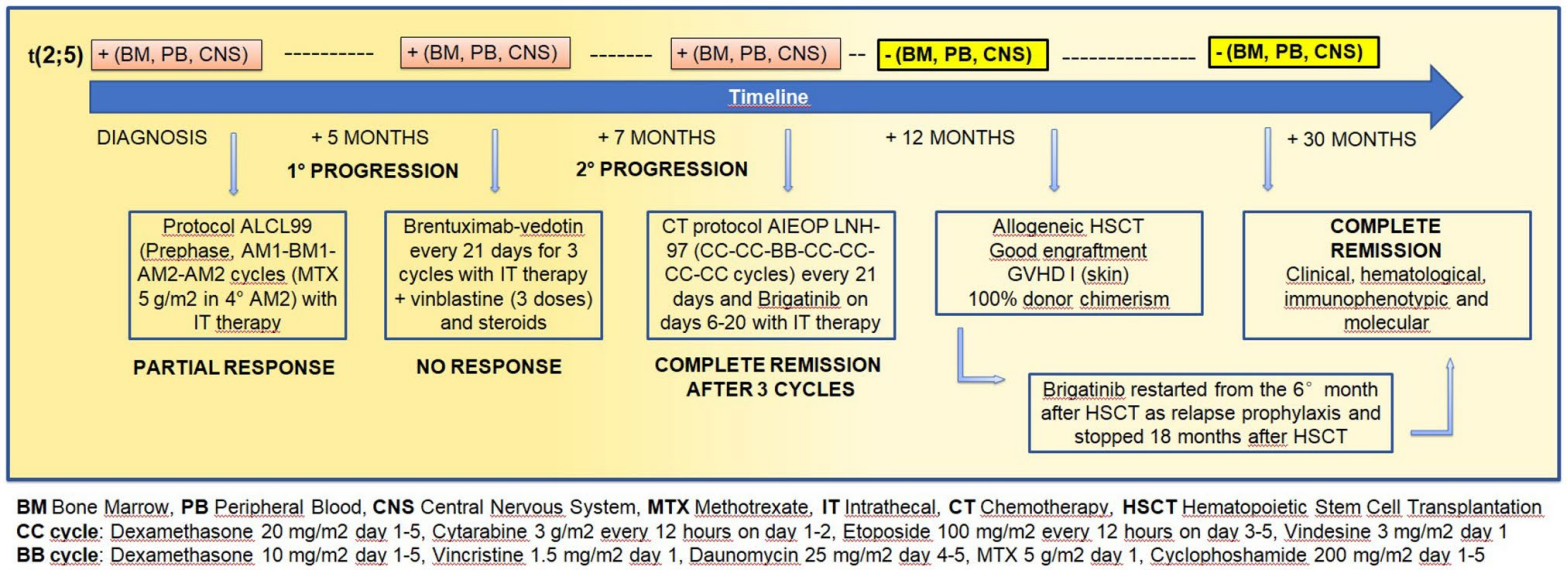
Summary of clinical history and therapeutic strategies adopted

A 13-year-old boy first presented with a history of persistent fever, marked asthenia and cervical lymphadenopathy without evidence of any other causative infection or inflammatory disorder. The assessment of bone marrow aspirate showed the presence of 18% of medium-large T lymphoid blasts with CD30 expression (immunophenotype at flow cytometry: CD 3 + -, CD8 + , CD4 + , CD5 −, CD2 + , CD7 + , CD56 −, CD57 −, HLA-DR + , CD16 −, TCR alpha/beta ± , CD13 +  +  −, CD 30 +  +  −) suggesting a lymphomatous condition. The search for *t*(2;5) translocation involving the ALK gene by PCR resulted positive both in peripheral blood and bone marrow samples. The ALK antibody titer was 1/2250 [9]. Lumbar puncture performed after the “prophase” chemotherapy with steroids showed the presence of 11% of an atypical lymphoid population CD8 + /CD4 + /CD5 − /HLA − DR + while cytomorphology was dubious for blasts. A total body PET/CT scan with 18F-FDG revealed an advanced disease with multiple supra and sub-diaphragmatic lymphadenopathy with high metabolism activity, and a diffuse bone uptake consistent with a lymphomatous disease. The final diagnosis of stage IV ALCL. Figure 1 summarizes the main clinical features presented during the course of his disease.

After a steroid-based treatment, the boy started frontline therapy according to the ALCL99 protocol including six courses of high-dose chemotherapy in association with intrathecal prophylaxis of central nervous system (CNS) disease [3, 10, 11].

Disease revaluation performed after the first four chemotherapy cycles (AM1, BM1, AM2, BM2) showed an unsatisfactory response, with persistence of multiple lymphadenopathies at PET/CT scan and the evidence of systemic disease at the immunophenotypic and molecular assessment of peripheral blood and bone marrow. The therapeutic strategy was then changed, with introduction of the anti-CD30 antibody–drug conjugate brentuximab-vedotin, in association with steroids and vinblastine as second-line therapy. After three courses of brentuximab-vedotin 21 days apart, the disease progressed further, with worsening of the known lymphadenopathies and the reappearance of atypical CD8 + /CD4 + /CD5 − /HLA-DR + cells in the cerebrospinal fluid.

Given the refractoriness of the disease to chemotherapy and immunotherapy and the CNS progression, the patient started a combination of high-dose chemotherapy based on the Italian Association of Pediatric Hematology and Oncology protocol for non-Hodgkin lymphoma (CC and BB cycles of the AIEOP LNH-97 protocol) [12] and the second-generation ALK-inhibitor brigatinib. The drugs included in the chemotherapy regimen are specified in Fig. 1. Brigatinib, unlike the first-generation ALK-inhibitor crizotinib, has the ability to pass the blood–brain barrier [13, 14]. This molecule was obtained for compassionate use after approval by the local Ethics Committee and the informed consent of the parents. Brigatinib was administered between chemotherapy courses that were scheduled with a 21-days interval (days 1–5), depending on the speed of myeloid recovery. It was started at a dose of 90 mg/day for the first week, then continued at 180 mg/day, and was well tolerated. After three chemotherapy courses (2 CC, 1 BB), a complete clinical, immunophenotypic and molecular remission was obtained. This result was confirmed by the disappearance of any pathologic uptake at the PET/CT scan. Meanwhile, the donor search identified an unrelated donor with a 9/10 HLA match. The patient bridged HSCT with three further courses of CC block chemotherapy (at a reduced dose) and brigatinib. He underwent allogeneic HSCT 12 months after diagnosis, after myeloablative conditioning regimen based on total body irradiation (TBI) and thiotepa. Brigatinib was withdrawn before HSCT.

GVHD prophylaxis was performed with one dose of cyclophosphamide of 50 mg/kg on day + 4 followed by cyclosporine and mycophenolate mofetil from day + 5. The patient engrafted early (day + 15 for neutrophil and day + 16 for platelets) and developed skin GVHD grade I on day + 23 responsive to a short course of steroids (1 mg/kg of prednisone). He was discharged home on day + 36 post-HSCT. On day + 46, he presented a flare of skin-gut-liver grade II GVHD, responsive to tacrolimus, prednisone, and two low doses of cyclophosphamide (day + 47 and day + 68), although the platelet count dropped to less than 50 × 10^9^/L for 2 months. Brigatinib, resumed at 2 months from HSCT as prophylaxis of relapse, was then withdrawn after a few days, and restarted 6 months after HSCT, once the platelet count had steadily increased to more than 100 × 10^9^/L. It was continued for 12 months and stopped 18 months after transplant. After 24 months from HSCT, the patient is in good clinical condition, full donor chimera, in complete hematologic and molecular remission, and enjoying a normal life.

### Summary of Published Cases of Treatment of ALCL Patients with ALK Inhibitors

The literature search was performed on the PubMed library accessed on 31 July 2022, using the keywords anaplastic large cell lymphoma, pediatric, crizotinib, alectinib, lorlatinib and brigatinib. Eight reports were identified describing the use of an ALK inhibitor in 65 patients (crizotinib 61, alectinib 3, lorlatinib 1). Table 1 shows the main demographic and clinical characteristics, response rate, outcome, main adverse effects, and status at the last follow-up.

**Table 1:** Summary of published experiences with a tyrosine kinase inhibitor in ALCL patients

Author (year)	Patients characteristics	Sex/age	ALK inhibitor	Outcome	Follow-up	Notes
Rindone (2022)	Retrospective r/r ALK + ALCL/NHL Previous treatment: CT lines: median 2 (range 1–6); BV: 6 patients; Auto-HSCT: 7 patients; Allo-HSCT: 1 patient	25 patients	Crizotinibmonotherapy 2 × 250 mg/day	1-month ORR 80%	31 months (range 0–140) 2-year PFS 57% 2-year OS58%	37% had AE but no patient withdrawn Alive patients are on crizotinib treatment
Verran (2022)	CNS Relapsed ALK + ALCL 3 months after 6 cycles of CHOEP	M, 20 year	Alectinib monotherapy, 2 × 600 mg/day	Complete clinical and radiological response after 3 months	Alive and well 24 months after starting treatment	Still on alectinib
Mellacheruvu(2022)	Lymphomatous meningitis Previous treatment: CHOP: 6 courses; BV: 9 courses; Auto-HSCT with BEAM; Crizotinib for11 months	M, 61 year	Lorlatinib, 100 mg/day	Complete clinical and radiological response after 11 months	Alive and well after 11 months	Short suspension of lorlatinib for AE, resumed at 50 mg/day
Zhang (2022)	IV stage ALCL Refractory to first line-CT	F, 10 years F, 13 years	Crizotinib with CT (BFM- R3/R4)	Complete clinical response	Alive and well	
Vanheeswijck(2021)	IV stage ALCL, Progression afterALCL99 CT (prophase and 3 courses) and ICM	M, 8 year	Crizotinib, 2 × 280 mg/m2/day for 2 weeks, then combined with Vinblastine 6 mg/m^2^ for 3 weeks	Partial response after 4 weeks of crizotinib	Progression with brain metastasis after 2 weeks from crizotinib suspension	Suspended for toxicity(neutropenia, bacteremia, mucositis, epidermal necrosis)
Shen (2021)	ALCL relapsed 5 months after stopping first line CT Persistence of ALK + PCR on bone marrow after 4 courses of CT Stage IV ALCL with persistence of CNS ALK + after 1 course of CT and 3 weeks of crizotinib	F, 11 year F, 22 months F, 11 year	Crizotinib 250 mg/day Alectinib 150 mg/day, initially combined with 2 courses of CT, then alone Alectinib,2 × 300 mg/day,initially alone then combined with 4 courses of CT	Complete response after 3 months Complete molecular response Complete molecular response on CNS after 2 weeks of alectinib	Alive and well after 25 months Alive and well 15 months after Alectinib Alive and well after 19 months from alectinib	Still on crizotinib Still on alectinib Still on alectinib
Tami (2018)	r/r ALCL, previous treatment: 3 ALCL99; 1 CHOP; 1 NHL-BFM	6 patients, 14.8–22.6 years	Crizotinib monotherapy, 2 × 280 mg/m^2^ for 2.5–23 courses of 28 days	Clinical and CT-PET remission followed by allogeneic HSCT	5 alive and well at median of 3.5 years after HSCT (range 0.4–4.5); 1 died after isolated CNS relapse	Crizotinb reduced in 2/6 patients for AE
Mosse (2017)	r/r ALCLafter 1–6 lines of CT, 2 after auto-HSCT,1 after BV	8 F, 18 M, 3.7–20.8 years	Crizotinib monotherapy2 × 165 mg/2/day (6 patients) and 2 × 280 mg/2/day (20 patients), 4–30 courses of 28 day	21 complete response; 2 Partialresponse; 3 stable disease	12 patients underwent allo-HSCT	AE neutropenia (33–70%) and alanine aminotransferase increase, 2 patients stopped crizotinib for AE

*CT* chemotherapy, *BV* Brentuximab-Vedotin, *Auto-HSCT* Autologous HSCT, *Allo-HSCT* Allogeneic HSCT, *ORR* Overall Response Rate, *OS* Overall Survival, *PFS* Progression Free Survival, *AE* Adverse Event

No report was found on brigatinib.

## Discussion

### ALK-Positive ALCL: Global Prognosis and Risk Factors For Treatment Failure

ALK-positive ALCL is considered a chemo-responsive disease with a probability of event-free survival of 70–75% with the current first-line protocols [2, 15]. On the other hand, the prognosis of patients who show refractoriness to first-line chemotherapy or relapse within 12 months after diagnosis is very poor, with a probability of survival ranging from 0 to 28% [10, 16–18]. The identification of these high-risk patients is still challenging, and is currently based on clinical factors such as:

- the visceral, bone marrow and CNS involvement at diagnosis;
- the demonstration by PCR for NPM-ALK of minimal disseminated disease (MDD) on bone marrow and peripheral blood at diagnosis;
- the persistence of minimal residual disease on bone marrow and peripheral blood during chemotherapy;
- the low titer of the anti-ALK antibody;
- more recently, the detection of micro-RNAs (miRNA) in exosomes on the peripheral blood of patients [3, 19, 20].

In a retrospective analysis of 128 patients, the combination of MDD and anti-ALK antibody titer allowed to stratify patients into three groups at high, intermediate, and low-risk of relapse and, in multivariate analysis, the high-risk biological characteristics and no common histology were both predictive of treatment failure [3, 20].

### New Treatment Options for Patients with High-Risk ALCL

The knowledge of the unfavorable clinical and biological profile for ALCL helps design new schemes of treatment, which include the early introduction of rescue therapy or combining chemotherapy with innovative targeted molecules. In the last decade, three types of new target drugs showed efficacy and safety against ALCL: the antibody–drug conjugate directed against the CD30 + antigen expressed on the surface of activated B and T lymphoma cells, the first and second generation ALK oncoprotein inhibitors in the context of ALK positive lymphomas, and the monoclonal antibodies against the PD-1 receptor and ligand. All these drugs have been used or experimented within different scenarios, such as first-line therapy in high-risk patients, salvage therapy in relapsed patients before HSCT, and salvage therapy after HSCT [2].

### ALK Inhibitors

ALK inhibitors are a group of tyrosine kinase inhibitors (TKI) targeting tumor cells expressing oncogenic mutations on the ALK. They act by inhibiting the permanently activated abnormal ALK protein, blocking downstream signaling pathways involved in tumoral growth. Over the years, the use of ALK inhibitors has been studied and implemented in particular in ALK-positive non-small-cell lung cancer (NSCLC), but represents a therapeutic option in a series of other tumors such as pediatric ALCL, inflammatory myofibroblastic tumor (IMT) and neuroblastoma, which can express different types of ALK oncogenic mutations [21].

Table 2 lists the main ALK inhibitors currently approved for use in clinical practice.

**Table 2:** ALK inhibitors currently approved for their use in clinical practice

ALK Inhibitor	Mechanism of action	Indications	Approval	Blood–brain barrier crossing	Main Adverse Effects
CRIZOTINIB	Competitive binding within the ATP-binding pocket of the target ALK Also inhibits cMET	Metastatic ALK + NSCLC Pediatric r/r ALCL and r/r IMT (approved > 1 y in US, > 6 y in Europe)	FDA: Aug 2011 EMA: Oct 2012	Insufficient	Vision problems GI symptoms Oedema Increased liver enzymes Neuropathy Prolonged QT
CERITINIB	Antagonist of the ALK tyrosine kinase receptor (inhibition of ALK mutated autophosphorylation) Also inhibits ROS1, IGF-1R and the insulin receptor	Metastatic ALK + NSCLC	FDA: Apr 2014 EMA: Jul 2017	Good	GI symptoms Increased liver enzymes Tiredness Anaemia Skin rash
ALECTINIB	Antagonist of the ALK tyrosine kinase receptor (inhibition of ALK mutated autophosphorylation) Can inhibit certain ALK mutations that follow treatment with crizotinib	Metastatic ALK + NSCLC	FDA: Dec 2015 EMA: Feb 2017	Good	Constipation Muscle pain Oedema
BRIGATINIB	Antagonist of the ALK tyrosine kinase receptor (inhibition of ALK mutated autophosphorylation) Multikinase inhibitor active also on EGFR kinase	Metastatic ALK + NSCLC	FDA: Apr 2017 EMA: Nov 2018	Good	Hyperglycaemia Hyperinsulinism Cytopenias GI symptoms Increased liver enzymes Myalgias and arthralgias
LORLATINIB	ATP-competitive inhibitor of the receptor tyrosine kinases, anaplastic lymphoma kinase (ALK) and C-ros oncogene 1 (Ros1)	Metastatic ALK + NSCLC	FDA: Nov 2018 EMA: May 2019	Good	Hypercholesterolemia Hypertriglyceridemia Oedema Peripheral neuropathy Weight gain Memory problems GI symptoms Arthralgias

*NSCLC* Non Small Cell Lung Carcinoma, *IMT* Inflammatory Myofibroblastic Tumor, *ALCL* Anaplastic Large Cell Lymphoma, *FDA* Food and Drug Administration, *EMA* European Medicines Agency

The vast majority (> 95%) of pediatric ALCL cases demonstrate overexpression of ALK, usually due to the classic (2;5) translocation of ALK with the nucleophosmin gene (NPM) [22].

### The Role of ALK Inhibitors in the Treatment of Patients with R/R ALCL

In a retrospective study on 18 R/R ALK-positive patients treated with crizotinib monotherapy and followed with PCR for ALK fusion transcript on peripheral blood after 4 and 12 weeks, progression was observed in 1 of 10 patients with negative PCR, versus 5 of 7 patients with positive PCR at the 4th-week assessment, and in 0 of 9 patients with negative PCR versus 3 of 4 patients with positive PCR at the 12th-week assessment. In that study, only 1 patient underwent allogeneic HSCT, because the aim was to assess the safety and efficacy of crizotinib as a rescue treatment for relapsed/refractory ALCL, and not as a bridge to HSCT [32].

Our case exemplifies the potentiality of new treatments for ALCL, and shows how an integrated rescue program based on the combination of chemotherapy, targeted therapy and HSCT can result in long-term survival and, eventually, in the definitive cure of the patient. This patient had all the worst clinical and biological characteristics which, up to a decade ago, would have brought to progression and death: a disseminated disease with bone marrow and CNS involvement, partial response and early progression after first-line chemotherapy, the persistence of MRD on bone marrow, peripheral blood, and CNS, refractoriness, and progression despite second-line therapy with brentuximab-vedotin [20, 23]. Although complete remission and long term survival have been reported in some patients who underwent allogeneic HSCT with active lymphoma, the presence of clinical disease or a positive MRD before HSCT are strong risk factors for relapse after HSCT [20, 23].

The decision to opt for the combination of high-dose chemotherapy and ALK inhibitor to achieve a clinical, and possibly, molecular remission was based on previous experience and literature data showing an advantage compared with chemotherapy alone or targeted therapy alone with crizotinib or brentuximab [2, 6, 24, 33–35]. Moreover, in vitro studies on ALCL cell lines and in vivo on mice showed that the combination of chemotherapy with crizotinib, a first-generation TKI, was more effective in controlling cell expansion and proliferation, inducing higher rates of apoptosis and inhibiting tumor growth [34].

Crizotinib is currently the only ALK-inhibitor approved for pediatric patients with R/R ALK + ALCL, while the second-generation ALK-inhibitors are still under investigation in this setting [14, 37]. This is the first reported pediatric case treated with brigatinib. Considering the long history of CNS disease, we decided to opt for this molecule, a second generation ALK inhibitor, instead of crizotinib, for its ability to penetrate through the blood–brain barrier, and its higher efficacy and lower risk of emerging resistance [14, 37, 38]. While there are no data on the use of brigatinib in pediatric ALK-positive tumours such as ALCL, IMT, neuroblastoma and glioma, data in adults affected by NSCLC showed superiority of brigatinib compared with crizotinib in terms of progression-free survival, response rate, intracranial tumor response rate, and death rate [37–40]. Given the refractoriness history of this patient and the difficulty to achieve clinical and molecular remission, we also decided to administer brigatinib for additional 12 months after allogeneic HSCT to prevent relapse. The use of maintenance therapy to target an undetectable minimal disease that is responsible for relapse after HSCT is adopted successfully in other types of diseases at high risk of relapse, such as acute myeloid leukemia, myelodysplastic syndrome, or non-Hodgkin lymphoma. This treatment was well tolerated.

### The Role of Allogeneic HSCT in the Treatment of Patients with R/R ALCL

Despite there not being a universal consensus, allogeneic HSCT is considered the best option to obtain or consolidate a long-term remission status in high-risk patients who relapsed at least once [2, 6, 8, 23–26]. This choice is particularly justifiable in patients with a history of MDD/MRD positivity on bone marrow or peripheral blood, because allogeneic HSCT allows the use of a tumor-free graft and exploits the graft versus lymphoma associated with donor-versus-recipient alloreactivity [7]. The most used preparative conditioning regimens are myeloablative, with or without total body irradiation, while the use of reduced conditioning regimens (RIC) to contain transplant-related mortality is still a matter of investigation [2, 6, 23, 24, 26–28]. In a series of 6 R/R ALCL patients who underwent allogeneic HSCT after obtaining a complete clinical and radiological remission with crizotinib monotherapy, 3 of 4 who were conditioned with a RIC presented mixed chimerism which required repeated donor lymphocyte infusions. One of these patients reverted to full-donor chimera but developed extensive chronic GVHD, while one of the other two patients relapsed on the CNS 3.6 years after HSCT and died [29]. Although allogeneic HSCT was effective in determining long-term disease-free survival also in patients transplanted with active lymphoma or relapsed after autologous HSCT [6, 24, 25], in analogy with relapsed acute lymphoblastic leukemia and Hodgkin lymphoma, it is reasonable to think that the remission status before HSCT plays a key role in obtaining the complete eradication of ALCL and long-term survival [29–31].

### ALK Inhibitors in ALCL: Limitations and Perspectives

The absolute number of patients diagnosed with ALCL, both in the pediatric and adult setting, is relatively low, leading to the need for well-coordinated multicenter trials with the aim of gradually fill the unmet medical needs in this field*.* Probably, the most relevant of these is to implement strategies to correctly identify those high risk patients who can benefit from the inclusion of ALK inhibitors and other target therapies as first-line therapies, with the aim of further improving the prognosis or, at least, of reducing the toxicity associated with conventional chemotherapy.

The Children’s Oncology Group (COG) Clinical Trial ANHL12P1 [11] is currently comparing the association between chemotherapy and brentuximab-vedotin or crizotinib in newly diagnosed stage II-IV ALCL. The results of the brentuximab-vedotin arm have already been published, showing a good toxicity profile, the disappearance of relapses during treatment and no significant interference on chemotherapy timing, while the results of the crizotinib arm are expected.

Brigatinib is actually in the process of being evaluated in a clinical trial in R/R ALCL [41].

On the model of NLCLC treatment, to have more than one ALK inhibitor available could represent a viable alternative for those patients who develop resistance to crizotinib, who are primarily resistant to it or who have CNS involvement. Most tumors eventually develop resistance through various mechanisms, such as compound-mutation (expressing 2–3 mutations simultaneously), activation of alternative pathways (i.e. MET pathway) and mutation in the ATP binding pocket. Brigatinib is a next generation inhibitor with activity against a broad spectrum of resistant ALK mutants. To overcome ALK inhibitor resistance, an interesting study is investigating possible alternative strategies such as the combination of ALK inhibitors and inhibitors of the tyrosine phosphatase SHP2 that directly regulate ALK phosphorylation and that seems to be overactivated in resistant ALK variants [42]. Finally, it remains to better understand for how long it is safe to administer an ALK inhibitor therapy and if it can effectively have a role as long term maintenance treatment after remission, also in the post-transplant setting.

## Conclusion

We presented a case of R/R ALCL in an adolescent patient who failed to obtain remission, and progressed despite conventional multiagent chemotherapy. The combination of brigatinib, high-dose chemotherapy and allogeneic HSCT allowed us to achieve a long-term clinical, radiological and molecular remission. This case shows the potential role of the combination of second-generation ALK inhibitors with chemotherapy and HSCT in R/R ALCL patients.

## Data Availability

Patient’s data are available in the institutional repository.
